# Bluetooth Hyperosmia: Chemosensory Variant of Delusional Somatic Symptom Disorder

**DOI:** 10.1192/j.eurpsy.2023.1443

**Published:** 2023-07-19

**Authors:** S. Kalita, D. Birwatkar, A. Hirsch

**Affiliations:** 1 Psychiatry; 2 Spartan Health Sciences University, Vieux Fort, Saint Lucia; 3 Psychiatry, Smell and Taste Treatment & Research Institute, Chicago, United States

## Abstract

**Introduction:**

Subjective hyperosmia, as a manifestation of belief of exposure to Bluetooth transmission, with testing demonstrating the absence of true hyperosmia, has not heretofore been described.

**Objectives:**

Correlation of Bluetooth transmission with subjective hyperosmia.

**Methods:**

This 53-year-old right-handed single woman presented with a 10-year history of increase in sensitivity to aroma and enhanced perception of smells upon exposure to Wi-Fi electromagnetic radiation. She noted an intensity-duration effect: with higher intensity and duration of Wi-Fi exposure, her sense of smell would escalate and persist: after a few hours of exposure, her smell would jump to a 190% of normal and last for two weeks. When she drives toward a metropolitan area, she can feel that the Wi-Fi is more intense and gets an electrical sensation like “I am an antenna”. Because of this, she refuses to use a cell phone or have Wi-Fi in her home.

**Results:**

Mental Status Examination: Able to recall 3 out of 4 objects in 3 minutes without reinforcement. Chemosensory Testing: Olfaction: Brief Smell Identification Test: 9 (normosmia). Alcohol Sniff Test: 8 (hyposmia). Gustation: Waterless Emperical Taste Test: brothy: 4/8 (hypogeusia), total: 46 (normogeusia).

**Conclusions:**

Nidus for such hyperosmic delusions may be a primary olfactory system disorder, with induction of ephaptic transmissions, causing intermittent phantosmia or otherwise misperceived odor, misattributed to the ambient environment. Paradoxically, such perceived hyperosmia may be due to a specific or isolated hyposmia or anosmia, the olfactory equivalent to monochromatic color blindness. The assignment of the source of the hyperosmia to that of Bluetooth is consistent with the zeitgeist of mistrust and paranoia of higher technology. Thus, the subjective hyperosmia would only occur when the patient perceives there was a kippage of radiation/ Bluetooth/ electromagnetic waves present, independent of these actually being present. This may be a form of expectation effect due to visual evidence (high tower wires); suggestion combined with subcultural group dynamics with belief in harm of such electromagnetic/Bluetooth waves, with distorted information recall and misattribution. Such group dynamics and shared misperceptions may act to fuel such a delusion as in the Mandela effect (French, 2018). This may represent the chemosensory equivalent of somatosensory amplification due to external intensification (Brascher, 2017). Perchance, this case represents not delusional hyperosmia, due to a functional psychiatric disorder, but rather has a neuroanatomic basis. Those with subjective hyperosmia and hypersensitivity to aromas have demonstrated hypertrophied gray matter volume in the posterior subregion of the right hippocampus, left precuneus, left superior frontal gyrus, and right hypothalamus (Han, 2020). In those with subjective hyperosmia, neurological investigation is warranted.

**Disclosure of Interest:**

None Declared

